# Low expression of *γ*-glutamyl hydrolase mRNA in primary colorectal cancer with the CpG island methylator phenotype

**DOI:** 10.1038/sj.bjc.6604346

**Published:** 2008-04-15

**Authors:** K Kawakami, A Ooyama, A Ruszkiewicz, M Jin, G Watanabe, J Moore, T Oka, B Iacopetta, T Minamoto

**Affiliations:** 1 Division of Translational and Clinical Oncology, Molecular and Cellular Targeting Translational Oncology Center, Cancer Research Institute, Kanazawa University, 13-1 Takara-machi, Kanazawa 920-0934, Japan; 2 Personalized Medicine Research Laboratory, Taiho Pharmaceutical Co., 224-2 Ebisuno, Hiraishi, Kawauchi-cho, Tokushima 711-0194, Japan; 3 Division of Tissue Pathology, Institute of Medical and Veterinary Science, Frome Road, Adelaide, South Australia 5000, Australia; 4 Department of General and Cardiothoracic Surgery, Kanazawa University Graduate School of Medical Science, 13-1 Takara-machi, Kanazawa 920-8641, Japan; 5 Colorectal Surgery Unit, Royal Adelaide Hospital, Adelaide, South Australia 5000, Australia; 6 School of Surgery and Pathology, University of Western Australia, 35 Stirling Highway, Nedlands, Western Australia 6009, Australia

**Keywords:** CIMP, GGH, promoter methylation, colorectal cancer

## Abstract

The CpG island methylator phenotype (CIMP+) in colorectal cancer (CRC) is defined as concomitant and frequent hypermethylation of CpG islands within gene promoter regions. We previously demonstrated that CIMP+ was associated with elevated concentrations of folate intermediates in tumour tissues. In the present study, we investigated whether CIMP+ was associated with a specific mRNA expression pattern for folate- and nucleotide-metabolising enzymes. An exploratory study was conducted on 114 CRC samples from Australia. mRNA levels for 17 genes involved in folate and nucleotide metabolism were measured by real-time RT-PCR. CIMP+ was determined by real-time methylation-specific PCR and compared to mRNA expression. Candidate genes showing association with CIMP+ were further investigated in a replication cohort of 150 CRC samples from Japan. In the exploratory study, low expression of *γ*-glutamyl hydrolase (*GGH*) was strongly associated with CIMP+ and CIMP+-related clinicopathological and molecular features. Trends for inverse association between GGH expression and the concentration of folate intermediates were also observed. Analysis of the replication cohort confirmed that GGH expression was significantly lower in CIMP+ CRC. Promoter hypermethylation of *GGH* was observed in only 5.6% (1 out of 18) CIMP+ tumours and could not account for the low expression level of this gene. CIMP+ CRC is associated with low expression of *GGH*, suggesting involvement of the folate pathway in the development and/or progression of this phenotype. Further studies of folate metabolism in CIMP+ CRC may help to elucidate the aetiology of these tumours and to predict their response to anti-folates and 5-fluorouracil/leucovorin.

Cancer is a disease with genetic and epigenetic abnormalities. Aberrant CpG island methylation is a common epigenetic alteration in a variety of malignancies (Jones, 2002). *De novo* methylation of CpG islands in promoter regions is believed to contribute to tumorigenesis by causing transcriptional silencing of tumour suppressor genes. Colorectal cancer (CRC) is one of the malignancies in which epigenetic changes have been extensively analysed. Research on clinical samples has shown that a subgroup of CRC shows concurrent hypermethylation of a large number of CpG islands. These have been termed CIMP+, for CpG island methylator phenotype (Toyota *et al*, 1999). CIMP+ tumours occur more frequently in the proximal colon of older patients and are associated with the microsatellite instability (MSI+) phenotype, tumour-infiltrating lymphocytes (TILs) and mutations in the *BRAF* oncogene (Hawkins *et al*, 2002; van Rijnsoever *et al*, 2002; Samowitz *et al*, 2005). Quantitative DNA methylation analysis using real-time techniques indicates that approximately 17% of CRC are CIMP+ (Ogino *et al*, 2006; Weisenberger *et al*, 2006; Iacopetta *et al*, 2007). A panel of five CpG island markers was recently proposed to standardise the definition of CIMP+ status (Weisenberger *et al*, 2006).

Although the existence of a CIMP+ CRC subgroup is evident, the aetiology of this phenotype is not well understood. We previously reported that CIMP+ was associated with elevated concentrations of the folate intermediates CH_2_FH_4_ and FH_4_ in CRC tissues (Kawakami *et al*, 2003). This suggests that folate metabolism may be an important factor in determining the DNA methylation status of primary CRC. Folate plays a major role in cellular homeostasis as a donor of one-carbon units for DNA methylation, protein methylation and nucleotide synthesis. Increased dietary folate intake and serum levels of folate show correlations with increased global DNA methylation levels in epidemiological studies (Pufulete *et al*, 2005b), animal models (Sohn *et al*, 2003) and clinical intervention studies (Pufulete *et al*, 2005a). Associations between dietary folate intake (van Engeland *et al*, 2003) or genetic variants in folate-metabolising enzymes (Paz *et al*, 2002) and CpG island hypermethylation in CRC have also been reported, although other workers have found less evidence for this (Curtin *et al*, 2007; Slattery *et al*, 2007). These observations suggest that folate metabolism, at least in part, can influence CpG island methylation and may therefore be involved in the development of CIMP+ CRC, although firm evidence for this is still lacking.

Two key metabolic pathways for methyl donor/one-carbon transfer reactions are the synthesis of folate and nucleotides. In the present study, we hypothesised that a specific expression pattern for folate- and nucleotide-metabolising enzymes occurs in CIMP+ CRC. Our rationale was that a distinctive gene expression signature may be associated with the aberrant methyl group metabolism of CIMP+ tumours, as evidenced by the frequent CpG island hypermethylation. To test this hypothesis, the mRNA expression levels of 17 genes with important roles in folate and nucleotide metabolism were measured by real-time RT-PCR in two series of primary CRC in which the CIMP+ status was determined by methylation-specific real-time PCR.

## MATERIALS AND METHODS

### Samples

For exploratory analysis of gene expression levels, tumour samples from a consecutive series of 114 CRC patients undergoing elective surgery at the Colorectal Unit of the Royal Adelaide Hospital in Australia were used. These samples were snap-frozen in liquid nitrogen within 20–40 min of resection and stored at −70 °C. DNA was extracted using a QIAamp DNA mini kit (Qiagen, Hilden, Germany), according to the manufacturer's protocol. RNA was obtained from the corresponding formalin-fixed and paraffin-embedded (FFPE) tissues. FFPE tissue blocks were reviewed for quality and tumour content, and 5-μm-thick sections were obtained. Sections were mounted on uncoated glass slides, deparaffinised in xylene, hydrated and stained with nuclear fast red (American MasterTech Scientific Inc., Lodi, CA, USA). Tumour cells were isolated by laser capture microdissection (PALM Microsystem; Leica, Wetzlar, Germany), according to the standard procedures (Bonner *et al*, 1997). RNA isolation after dissection was performed according to a proprietary procedure (Response Genetics Inc., US patent no. 6248535). We have previously measured the concentrations of the folate intermediates CH_2_FH_4_ and FH_4_ (Kawakami *et al*, 2003) and analysed for *BRAF* V600E mutation (Iacopetta *et al*, 2006) in this tumour series. Approval of this project was obtained from the IMVS Human Research Ethics Committee.

For the validation tumour set, 150 primary CRC samples from patients undergoing surgical treatment at Kanazawa University Hospital in Japan were used. Tumour was dissected manually from FFPE archival tissue sections of 10 μm thickness. After deparaffinisation using xylene and ethanol, genomic DNA was isolated using a QIAamp DNA mini kit. RNA was obtained from the manually dissected FFPE samples using the same method as for the Australian CRC series. Approval of this project was obtained from the Ethics Committee of Kanazawa University School of Medicine.

### Real-time RT-PCR and immunohistochemistry

Complementary DNA was prepared as described previously (Lord *et al*, 2000). Quantification of the genes of interest (Table 1) and an internal reference gene (*ACTB*) was conducted using a fluorescence-based real-time detection method (ABI PRISM 7700 Sequence Detection System (TaqMan); Perkin-Elmer Applied Biosystems, Foster City, CA, USA), as previously described (Gibson *et al*, 1996; Dziadziuszko *et al*, 2006). Gene expression values were expressed as ratios (differences between *C*_t_ values) between the gene of interest and an internal reference gene (*ACTB*). Primer and probe sequences used in this study are listed in Supplementary Table 1.

For the validation study with Japanese CRC samples, different primer sets for *ECGF1*, *γ*-glutamyl hydrolase (*GGH*), *RRM2* and *ACTB* (Supplementary Table 1) were used with SYBR Premix Ex Taq (TaKaRa Bio, Otsu, Japan) and following the protocol provided by the manufacturer using ABI PRISM 7700 Sequence Detection System. The quantity of mRNA was expressed as the ratio of the expression level between each test mRNA and *ACTB* mRNA.

Protein expression of GGH in tumour tissues was examined by immunohistochemistry for selected samples from the Japanese CRC cohort. The avidin–biotin–peroxidase complex method with chicken polyclonal antibody (IgY) to human GGH (diluted 1 : 100; GenWay Biotech, San Diego, CA, USA) and biotinylated rabbit anti-chicken IgY (diluted 1 : 200; Open Biosystems, Huntsville, AL, USA) was used following microwave antigen retrieval of paraffin sections, as described previously (Ougolkov *et al*, 2002).

### Methylation analysis

Promoter methylation was evaluated for the CIMP panel of markers comprising *CACNA1G*, *IGF2*, *NEUROG1*, *RUNX3* and *SOCS1*, where PMR (percentage methylated reference) values were derived using the *ALU* normalisation control reaction (Weisenberger *et al*, 2006). Simultaneous hypermethylation (PMR ⩾10) of 3 or more of these 5 markers was considered to represent CIMP+. Promoter hypermethylation of G*GH* was analysed as previously described (Cheng *et al*, 2006). Sperm DNA and fully methylated DNA by *SssI* methylase (NewEngland Biolabs, Ipswich, MA, USA) were used as unmethylated and methylated control samples, respectively.

### Statistical analysis

Because mRNA expression levels did not show normal distribution, the results were expressed as median values (25th to 75th percentiles) in tables or boxplots. Non-parametric models were used for univariate analyses. The Mann–Whitney *U*-test was used to compare mRNA expression levels between two categorical variables. Correlations between mRNA expression and the concentration of folate intermediates were analysed by Spearman's rank test. A multivariate stepwise logistic regression approach was used to select genes whose mRNA expression was significantly related to CIMP status. All *P*-values shown are two tailed, with *P*<0.05 taken as significant.

## RESULTS

### Associations between mRNA expression levels for folate- and nucleotide-metabolising enzymes and CIMP+ or CIMP+-related features

RT-PCR assays were conducted for 17 genes in 114 colorectal tumour samples from Australia. The assays were performed in triplicate for RT samples and in a single assay for non-RT controls, resulting in 1938 mRNA measurements by 7752 assays. The non-RT control reaction was positive in 31 measurements and the coefficient of variance was high among triplicate assays in four measurements. These were deemed as ‘no result’. In all, 1903 out of 1938 (98.2%) real-time RT-PCR measurements were successful using RNA derived from laser capture microdissected FFPE tumour tissues. CIMP+ was found in 18 out of 114 (15.8%) CRC samples.

**Figure 1:**
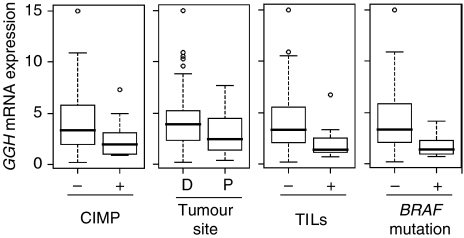
*γ*-Glutamyl hydrolase mRNA expression according to the CIMP status, tumour site, TILs and *BRAF* mutation status in an Australian CRC cohort was shown by boxplot. The level of *GGH* mRNA expression was significantly different between all dichotomised variables (Mann–Whitney *U*-test; CIMP, *P*=0.013; tumour site, *P*=0.021; TILs, *P*=0.001; *BRAF* mutation, *P*=0.002).

Cluster analysis did not reveal a distinctive mRNA expression profile associated with CIMP+ (data not shown). In univariate analysis (Mann–Whitney *U*-test), *GGH* expression was significantly lower in CIMP+ than CIMP− CRC, whereas the expression of *DCK*, *DPYD*, *ECGF1*, *MTHFR* and *RRM2* was all higher in CIMP+ (Table 2). Multivariate analysis using a logistic regression model showed that *GGH* expression (odds ratio 0.70, 95% CI: 0.51–0.95, *P*=0.023) and *RRM2* expression (odds ratio 1.25, 95% CI: 1.04–1.49, *P*=0.015) were associated with CIMP+ (*P*=0.008). Univariate analysis (Mann–Whitney *U*-test) showed that *ECGF1* and *GGH* expressions were strongly associated with the CIMP+ features of proximal tumour site, TILs and *BRAF* mutation (Table 3 and Supplementary Table 2). *γ*-Glutamyl hydrolase expression was lower, whereas *ECGF1* was higher in tumour with these CIMP+ features. The analyses showed that low expression of *GGH* was consistently associated with CIMP+ and CIMP+-related features (Figure 1). The high expression levels of *RRM2* and *ECGF1* also showed strong associations with CIMP+ and CIMP+-related features, respectively.

Finally, mRNA expression was compared to the concentrations of the folate intermediates CH_2_FH_4_ and FH_4_ in these CRC tissues (Table 4). None of the genes examined showed significant correlation with the concentrations of these intermediates, although high *RFC1* expression was significantly correlated with low concentrations of FH_4_ (Spearman's *ρ*=−0.205, *P*=0.046). Trends for negative correlation between *GGH* expression and CH_2_FH_4_ and FH_4_ concentrations were observed (Spearman's *ρ*=−0.200, *P*=0.053 and Spearman's *ρ*=−0.180, *P*=0.083, respectively). The above exploratory analyses suggest that low *GGH* mRNA expression is a candidate CIMP+ molecular signature, possibly through its involvement in folate metabolism.

### Validation of *GGH* downregulation in CIMP+ CRC

**Figure 2:**
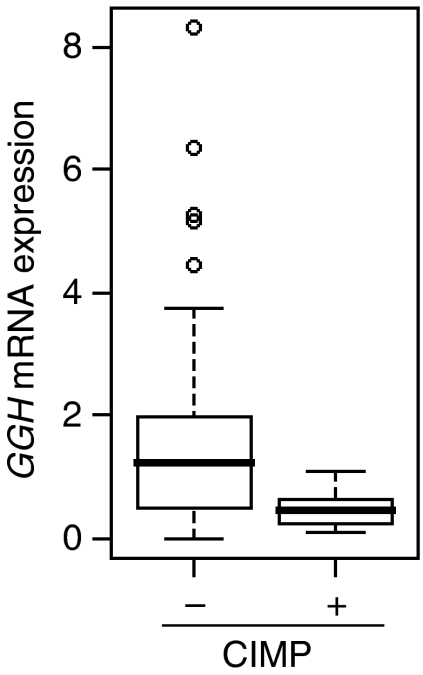
*γ*-Glutamyl hydrolase mRNA expression according to the CIMP status in a Japanese CRC cohort used for validation was shown by boxplot. Lower *GGH* expression in CIMP+ compared to CIMP− CRC was confirmed (Mann–Whitney *U*-test; *P*=0.0012).

A validation study was conducted using 150 primary CRC samples from a Japanese cohort of patients. *γ*-Glutamyl hydrolase, *ECGF1* and *RRM2* were selected as candidates for further study because the expression of these genes was consistently associated with CIMP+ and/or CIMP+-related features in the Australian CRC series. Only 14 out of 150 (9.3%) of the Japanese CRC samples were found to be CIMP+ compared to 15.8% of the Australian tumours (*P*=0.11 in *χ*^2^ test). A random selection of CIMP− CRC (*n*=79) and all 14 CIMP+ CRC samples from the Japanese cohort were subjected to RT-PCR analysis of *GGH*, *ECGF1* and *RRM2* expression. The results confirmed the previous result that *GGH* mRNA expression was significantly lower in CIMP+ CRC samples from a separate tumour series (*P*=0.0012, Figure 2). No significant associations were observed between CIMP+ and either *ECGF1* or *RRM2* mRNA expression.

**Figure 3:**
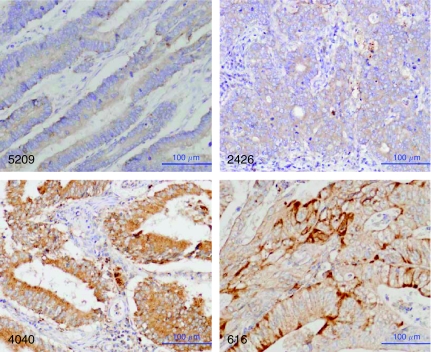
Immunohistochemical staining of GGH in CRC tissues. *γ*-Glutamyl hydrolase protein was not detectable in tumour cells in case Nos. 5209 and 2426, in which the *GGH* mRNA levels were 0.01 and 0.06, respectively. Expression of GGH was observed in tumour cells in case Nos. 4040 and 616, in which the *GGH* mRNA levels were 3.73 and 8.29, respectively.

To further examine whether the mRNA level reflects GGH protein expression, selected paraffin tissues of Japanese CRC were immunostained using polyclonal antibody to human GGH. *γ*-Glutamyl hydrolase protein was not detectable or was weakly expressed in four samples with low mRNA levels (0.01, 0.06, 0.28 and 0.32), whereas much stronger expression was observed in five samples with high mRNA levels (3.25, 3.50, 3.73, 6.33 and 8.29). Representative cases are shown in Figure 3. The results indicate an association between levels of *GGH* mRNA and its protein expression.

### *GGH* promoter methylation is not a cause of *GGH* downregulation in CIMP+ CRC

**Figure 4:**
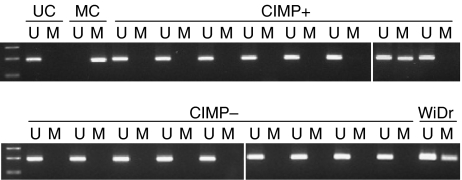
Methylation-specific PCR analysis of the *GGH* promoter. Promoter methylation of *GGH* was analysed using unmethylated DNA-specific primer sets (U) and methylated DNA-specific primer sets (M). Representative results using 18 samples of CIMP+ and 20 randomly selected samples of CIMP− tumours are shown. Only one sample, a CIMP+ tumour, showed *GGH* promoter hypermethylation. Sperm DNA and fully methylated sperm DNA produced with *SssI* methylase were used for unmethylated control (UC) and methylated control (MC), respectively. DNA from the colon cancer cell line WiDr was also used as a positive control.

A recent study in leukaemia found that hypermethylation of the *GGH* promoter was associated with silencing of *GGH* gene expression (Cheng *et al*, 2006). The above exploratory analyses showing that CIMP+ CRC samples have low *GGH* mRNA expression levels also raise this possibility. Methylation of the *GGH* promoter was therefore analysed in 18 CIMP+ tumours and in 20 randomly selected CIMP− tumours from the Australian CRC cohort. Only one CIMP+ tumour (5.6%) showed hypermethylation of the *GGH* promoter (Figure 4), indicating that it does not play a major role in downregulating the mRNA expression of this gene in CRC.

## DISCUSSION

**Figure 5:**
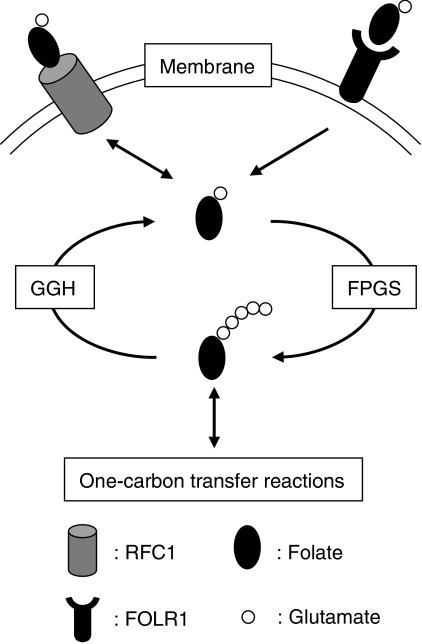
Simplified representation of folate transport and polyglutamylation reactions within the cell. RFC1 is ubiquitously expressed in epithelial cells and plays a role as a major transport system for folates. FOLR1 is anchored to cell membranes and transports folates via an endocytotic process. Intracellular monoglutamyl folate is converted to the polyglutamate form by FPGS, whereas the polyglutamate chains are removed by GGH.

In this study, we explored the possibility that genes involved in folate and nucleotide metabolism have a distinct mRNA expression signature in CIMP+ CRC. Although no clear expression pattern was found for the 17 genes analysed, low *GGH* expression was observed in two independent series of CIMP+ CRC and could therefore play a role in the development of this phenotype. In the Australian CRC cohort, the mRNA expression levels for *GGH* and *RRM2* were shown by univariate and multivariate analyses to be significantly associated with CIMP+ CRC. In addition, the mRNA expression of *GGH* and *ECGF1* was associated with characteristic clinicopathological and molecular features of CIMP+, including proximal tumour site, TILs and *BRAF* mutation. Moreover, the concentrations of two folate intermediates, CH_2_FH_4_ and FH_4_, showed trends for association with *GGH* mRNA expression. As might be predicted from the function of GGH in hydrolysing glutamated folates and allowing escape from the cell (Figure 5), low *GGH* expression was associated with higher folate concentrations.

Overall, the results of the exploratory study on the Australian CRC cohort provided evidence that low expression of *GGH* mRNA was associated with CIMP+ and with CIMP+-related features. This led us to conduct a further study using an independent cohort of primary CRC from Japan and in which we confirmed the relationship between low *GGH* mRNA expression and CIMP+ status. The frequency of CIMP+ was lower among Japanese CRC (9.3%) than that among Australian CRC (15.8%). Although this difference did not reach statistical significance, it suggests that dietary, environmental and genetic differences between these two populations could influence the frequency of the CIMP+ subgroup as a proportion of total CRC. Nevertheless, low expression of *GGH* mRNA was a consistent finding in both CIMP+ cohorts. Although a recommended panel of markers was used here to define CIMP+ (Weisenberger *et al*, 2006), the *GGH*/CIMP+ association was also found using a different CpG island panel comprising of *MLH1*, *p16 (INK4A)*, *TIMP3* and *p14 (ARF)* (data not shown).

The present results suggest that low *GGH* mRNA expression may play a role in the development and/or progression of CIMP+ CRC. A possible explanation for this is the role played by GGH in regulating intracellular folate levels (Figure 5). Monoglutamyl folate is transported into mammalian cells mainly by FOLR1 and RFC1 (Matherly and Goldman, 2003). Intracellular monoglutamyl folate is converted to the polyglutamate form by FPGS (Qi *et al*, 1999), whereas the polyglutamate chains are removed by GGH (Schneider and Ryan, 2006). Polyglutamate forms of folate are more strongly retained within the cell and are a better substrate for intracellular folate-dependent enzymes than the monoglutamate form. Therefore, low GGH expression would be expected to lead to a higher concentration of polyglutamated folate because of better retention in the cell. In agreement with this, we observed trends for an inverse correlation between *GGH* expression and the concentrations of folate intermediates CH_2_FH_4_ and FH_4_ (Table 4). *FOLR1* and *FPGS* mRNA expression were not associated with the concentrations of these folate intermediates; however, the increased expression of *RFC1* was significantly correlated with low concentrations of FH_4_. These results suggest that *GGH* expression plays a role in regulating the intracellular folate level in CRC tissues, although other factors such as *RFC1* expression are also likely to be involved.

We previously reported that frequent CpG promoter hypermethylation was associated with high folate levels in CRC (Kawakami *et al*, 2003). A recent study also found that the level of *p16* (*INK4A*) promoter methylation in the normal colonic mucosa of older mice increased following folate supplementation (Keyes *et al*, 2007). Together, the above results suggest that low *GGH* expression may be linked to increased promoter methylation in CIMP+ tumours by causing elevation of the folate concentration. An alternate explanation involving transcriptional silencing of *GGH* by promoter methylation was excluded by the finding that only 5.6% of CIMP+ tumours showed *GGH* hypermethylation (Figure 4). It is unknown whether low *GGH* expression and its link with high tissue folate concentrations play a causal or even supportive role in the development of CIMP+ CRC. Further studies are required in which *GGH* expression, folate status and CpG island methylation are evaluated in normal colonic tissue as well as in the proposed precursor lesion for CIMP+, the so-called serrated adenoma or hyperplastic polyp (Jass, 2006). The mechanism(s) by which *GGH* expression is regulated in both normal and malignant colorectal tissue also warrants further investigation. Apart from two studies that found *GGH* expression was increased in CRC compared to adjacent normal colonic mucosa (Odin *et al*, 2003; Kidd *et al*, 2005), no other work has been published in this area.

In addition to possible implications for the aetiology of CIMP+ CRC, the current findings are also relevant for the response of CRC to inhibitors of dihydrofolate reductase and thymidylate synthase (TS), both of which are key enzymes in nucleotide synthesis. The growth inhibitory effect of anti-folates such as methotrexate (MTX) and raltitrexed depends upon the polyglutamylation state of these agents (Barnes *et al*, 1999). Methotrexate is transported into cells using the same mechanism as that for folates and is also better retained following polyglutamylation. High GGH activity has been associated with the resistance of tumour cell lines to MTX via shortening of polyglutamate chains and consequently a lower intracellular drug concentration and less inhibition of dihydrofolate reductase and TS (Rhee *et al*, 1993; Barnes *et al*, 1999). Raltitrexed, a specific inhibitor of TS, is also polyglutamylated and its antitumour activity correlates with the amount of polyglutamylated drug inside the cells (Takemura *et al*, 1996). The importance of polyglutamylation in the antitumour activity suggests that CIMP+ CRC might have higher sensitivity to these anti-folates because of low *GGH* expression in this subtype of CRC. Neither MTX nor raltitrexed is widely used in chemotherapy for CRC. However, these anti-folates might be of clinical use for tailored chemotherapy.

In contrast to above-mentioned anti-folates, 5-fluorouracil (5-FU) and leucovorin have been key drugs for the chemotherapy of CRC. 5-Fluorouracil is thought to exert its major cytotoxic activity by inhibiting TS. It does this by forming a stable ternary complex between 5,10-methylenetetrahydrofolate, TS and fluoro-dUMP, the metabolite of 5-FU (Longley *et al*, 2003). Leucovorin, also known as folinic acid, increases the activity of 5-FU by raising the intracellular levels of 5,10-methylenetetrahydrofolate and thereby prolonging the inhibition of TS. 5,10-Methylenetetrahydrofolate is also better retained following polyglutamylation (Radparvar *et al*, 1989), and this is critical for the antitumour activity of 5-FU even when this folate intermediate is present at relatively high concentrations (Romanini *et al*, 1991). Therefore, supplementation of 5-FU with leucovorin may be more effective in CIMP+ compared to CIMP− CRC because the low GGH levels would better allow the retention and modulatory action of 5,10-methylenetetrahydrofolate. An earlier study did indeed find that adjuvant treatment with 5-FU/leucovorin conferred more benefit to CIMP+ tumours in stage III CRC (van Rijnsoever *et al*, 2003). Two more recent studies reported that CIMP+ CRC was associated with poor survival in advanced CRC treated with 5-FU-based chemotherapy (Ogino *et al*, 2007; Shen *et al*, 2007). This may, however, be a reflection of the prognostic rather than predictive value of CIMP+. Moreover, the regimens used in the two studies of advanced CRC included not only 5-FU alone but also in combination with other chemotheraputic agents such as IFNα-2a and irinotecan. Further prospective studies are needed to test whether CIMP+ is a predictive marker for response to 5-FU/leucovorin in CRC. These may allow chemotherapy regimens to be tailored according to the CIMP+ status, leading to more effective cancer treatment.

The present study investigated 17 genes involved in folate and nucleotide metabolism. Low expression of *GGH* was one of the features associated with CIMP+ CRC; however, it was not a specific marker for this phenotype because many CIMP− tumours also showed low expression of this gene. The aberrant promoter methylation observed in CIMP+ CRC is likely to be a multistep phenomenon that involves many factors in addition to folate metabolism and could include, for example, the expression levels of methyltransferases and histone deacetylase. Some of these factors may be revealed by array-based transcriptome analysis of CIMP+ and CIMP− CRC tissues. Although no study to date has addressed this issue, a few reports have described the mRNA expression profile of the closely associated MSI+ phenotype in CRC (Banerjea *et al*, 2004; di Pietro *et al*, 2005; Lanza *et al*, 2007). Interestingly, each of these studies showed that *GGH* mRNA expression was lower in MSI+ compared to MSI− CRC. Because of the strong concordance between MSI+ and CIMP+ in population-based CRC cohorts (Hawkins *et al*, 2002; van Rijnsoever *et al*, 2002; Samowitz *et al*, 2005; Ogino *et al*, 2006; Weisenberger *et al*, 2006), the results from these independent, array-based studies confirm the current results obtained using RT-PCR and two separate CIMP+ CRC series. Together, the studies provide strong evidences of low *GGH* expression in MSI+ and/or CIMP+ CRC. The three array-based studies did not show consistent association between expression of the other genes analysed in current study with MSI status. However, two of them (Banerjea *et al*, 2004; di Pietro *et al*, 2005) demonstrated higher expression of *TYMS* in MSI+ compared to MSI− CRC. Our result on *TYMS* expression between CIMP+ and CIMP− CRC did not support this association (*P*=0.099, Table 2). *TYMS* might differently express in MSI+ compared to CIMP+ CRC, requiring further study to establish molecular difference between MSI+ and CIMP+ CRC.

In conclusion, the present study is the first to investigate the expression of genes involved in folate and nucleotide metabolism in relation to CIMP+ CRC. This tumour phenotype is associated with low expression of *GGH*, suggesting involvement of the folate pathway in its development and/or growth. Further studies of folate metabolism in CIMP+ CRC, premalignant precursors and normal colonic mucosa may help to elucidate the aetiology of these tumours. A better understanding of the role of folate metabolism in DNA methylation may also lead to tailored chemotherapy that employs anti-folates, 5-FU/leucovorin and the use of CIMP+ markers.

## Figures and Tables

**Table 1:** Folate- and nucleotide-metabolising genes analysed in this study

**Gene symbol**	**Gene name**	**GenBank accession no.**
*CDA*	cytidine deaminase	NM_001785
*DCK*	deoxycytidine kinase	XM_003471
*DCTD*	dCMP deaminase	NM_001921
*DHFR*	dihydrofolate reductase	NM_000791
*DPYD*	dihydropyrimidine dehydrogenase	NM_000110
*DUT*	dUTP pyrophosphatase/deoxyuridine triphosphate nucleotidohydrolase	U90223
*ECGF1*	endothelial cell growth factor 1 (platelet-derived)/thymidine phosphorylase	M63193
*FOLR1*	folate receptor 1/folate receptor alpha	NM_016730
*FPGS*	folylpolyglutamate synthetase	M98045
*GGH*	gamma-glutamyl hydrolase	NM_003878
*MTHFD1*	methylene tetrahydrofolate dehydrogenase 1	NM_005956
*MTHFR*	methylene tetrahydrofolate reductase	NM_005957
*RFC1*	reduced folate carrier 1	NM_003056
*RRM1*	ribonucleotide reductase M1 subunit	X59543
*RRM2*	ribonucleotide reductase M2 subunit	NM_001034
*TYMS*	thymidylate synthase	NM_001071
*UMPS*	uridine monophosphate synthetase/orotate phosphoribosyl transferase	XM_050552

Gene symbol is based on the HUGO Gene Nomenclature Committee (http://www.genenames.org/index.html).

**Table 2:** Associations between mRNA expression and CIMP status in CRC from an Australian cohort

	**mRNA expression level**		
**Gene symbol**	**CIMP+**	**CIMP−**	***P*-value**
*CDA*	3.44 (1.79–5.05)	2.08 (0.92–4.28)	0.131
*DCK*	2.78 (2.57–3.22)	2.49 (1.67–3.00)	0.025
*DCTD*	4.21 (3.52–5.21)	4.04 (3.00–5.24)	0.403
*DHFR*	4.67 (3.52–5.37)	3.72 (2.84–5.38)	0.129
*DPYD*	0.48 (0.32–0.76)	0.32 (0.24–0.48)	0.025
*DUT*	123.8 (53.6–196.8)	116.2 (68.1–166.2)	0.828
*ECGF1*	4.76 (3.16–7.08)	2.71 (1.93–4.01)	0.001
*FOLR1*	0.00 (0.00–0.07)	0.10 (0.00–0.43)	0.060
*FPGS*	0.77 (0.56–0.95)	0.70 (0.52–0.85)	0.458
*GGH*	1.97 (1.04–3.06)	3.31 (1.98–5.69)	0.013
*MTHFD1*	4.46 (3.76–5.11)	3.84 (2.90–5.08)	0.197
*MTHFR*	1.20 (0.97–1.40)	0.91 (0.65–1.34)	0.044
*RFC1*	2.97 (2.29–4.30)	2.91 (1.99–3.92)	0.923
*RRM1*	1.02 (0.84–1.29)	0.96 (0.65–1.27)	0.265
*RRM2*	6.96 (4.60–7.70)	3.51 (2.27–6.03)	0.004
*TYMS*	3.32 (2.48–5.81)	2.99 (1.91–4.24)	0.099
*UMPS*	1.17 (1.07–1.40)	1.29 (0.96–1.73)	0.660

CIMP=CpG island methylator phenotype; CRC=colorectal cancer.

mRNA expression levels are shown as median (25th to 75th percentiles).

The Mann–Whitney *U*-test was used for statistical analysis.

**Table 3:** Associations between mRNA expression and clinicopathological and molecular features in CRC from an Australian cohort

	**Tumour site**		**TILs**		***BRAF* mutation**	
**Gene symbol**	**Proximal**	**Distal**	**Present**	**Absent**	**Present**	**Absent**
*CDA*	2.08	2.42	2.06	2.21	3.81	2.12
*DCK*	2.55	2.59	2.90	2.53^*^	2.70	2.54
*DCTD*	3.97	4.28	4.39	4.07	4.01	4.08
*DHFR*	3.74	4.39	4.52	3.78	4.75	3.90
*DPYD*	0.36	0.30	0.62	0.32^*^	0.59	0.32
*DUT*	123.2	117.2	166.3	112.7	126.3	114.6
*ECGF1*	3.44	2.50^*^	5.29	2.85^**^	6.73	2.96^**^
*FOLR1*	0.00	0.16^**^	0.15	0.08	0.00	0.08
*FPGS*	0.67	0.73	0.76	0.70	0.73	0.70
*GGH*	2.44	3.91^*^	1.38	3.35^**^	1.38	3.33^**^
*MTHFD1*	3.94	4.06	4.15	3.98	4.68	3.87
*MTHFR*	1.12	0.85	1.28	0.94	1.32	0.94^*^
*RFC1*	2.68	3.16^*^	2.70	3.00	2.70	3.02
*RRM1*	0.94	0.99	0.99	0.95	1.07	0.96
*RRM2*	4.36	4.28	5.33	3.91	7.01	3.91^*^
*TYMS*	3.35	2.69	4.74	2.64^**^	4.48	2.99^*^
*UMPS*	1.14	1.36^*^	1.13	1.29	1.13	1.30

CRC=colorectal cancer; TILs=tumour-infiltrating lymphocytes.

Median mRNA expression levels are shown.

The Mann–Whitney *U*-test was used for statistical analysis. ^*^P<0.05; ^**^P<0.01.

Data presented with median (25th to 75th percentiles) and *P*-value are available in Supplementary Table 2.

**Table 4:** Associations between mRNA expression level and the concentration of folate intermediates in CRC from an Australian cohort

	**CH**_**2**_ **FH**_**4**_		**FH**_**4**_	
**Gene symbol**	**Spearman's *ρ***	***P*-value**	**Spearman's *ρ***	***P*-value**
*CDA*	0.205	0.063	0.154	0.163
*DCK*	0.058	0.583	0.056	0.599
*DCTD*	−0.042	0.684	0.024	0.817
*DHFR*	−0.081	0.448	−0.145	0.174
*DPYD*	0.020	0.845	0.010	0.924
*DUT*	−0.022	0.834	−0.022	0.834
*ECGF1*	0.062	0.559	0.043	0.686
*FOLR1*	−0.136	0.190	−0.073	0.481
*FPGS*	−0.025	0.807	0.023	0.822
*GGH*	−0.200	0.053	−0.180	0.083
*MTHFD1*	−0.014	0.856	−0.033	0.752
*MTHFR*	−0.028	0.784	0.037	0.720
*RFC1*	−0.159	0.123	−0.205	0.046
*RRM1*	0.039	0.707	0.006	0.952
*RRM2*	0.107	0.303	0.046	0.661
*TYMS*	0.076	0.471	−0.018	0.864
*UMPS*	−0.121	0.246	−0.050	0.630

CRC=colorectal cancer.

Spearman's rank correlation test was used for analyses.

Spearman's *ρ* and *P*-value are presented for each analysis of correlations.
