# Supplementary material for: Low expression of γ-glutamyl hydrolase mRNA in primary colorectal cancer with the CpG island methylator phenotype
Source: Br J Cancer. 2008 Apr 15;98(9):1555–61. doi: 10.1038/sj.bjc.6604346 (PMC2391094; doi:10.1038/sj.bjc.6604346)
Supplement: Supplementary Tables [file 6604346x1.doc]

**Supplementary Table 1.** Primers used for real-time RT-PCR

Genes forward primer probe reverse primer

for Australian CRC series

CDA AAAGGGTGCAACATAGAAAATGC TGCTACCCGCTGGGCATCTGTG CTGGATAGCGGTCCGTTCA

DCK GACTGGCATGACTGGATGAA TCCATCCAATTCAAGGCTTTGGCC TCTCTGGAGTGGCTTGAAGA

DCTD GCAGAGAATAAGCTGGACACCAA TACCCGTACGTGTGCCATGCGGA TTTTTGTTCATGATGGCATTCAG

DHFR GTCCTCCCGCTGCTGTCA TTCGCTAAACTGCATCGTCGCTGTGTC GCCGATGCCCATGTTCTG

DPYD AGGACGCAAGGAGGGTTTG CAGTGCCTACAGTCTCGAGTCTGCCAGTG GTCCGCCGAGTCCTTACTGA

DUT GTCTCCTCGCTCGCCTTCT TTCAGAGCAGGGCATGGCAGAGC GGTGAAATGGCGGGTGTCT

ECGF1 CCTGCGGACGGAATCCT CAGCCAGAGATGTGACAGCCACCGT GCTGTGATGAGTGGCAGGCT

FOLR1 GAAGATTGTCGCACCTCCTACAC CCCTTGTGCCAGTTGCTCTTGCA CGCACTTGTTAAACCCTGAAGTC

FPGS GGCTGGAGGAGACCAAGGAT CAGCTGTGTCTCCATGCCCCCCTAC CATGAGTGTCAGGAAGCGGA

GGH GCGAGCCTCGAGCTGTCTA ACCCCACGGCGACACCGC AATATTCCGATGATGGGCTTCTT

MTHFD1 CGTGGGCAGCGGACTAA CGCCAGCAGAAATCCTGAACGG CCTTATTTGCGCGGAGATCT

MTHFR CGGGTTAATTACCACCTTGTCAA TGAAGGGTGAAAACATCACCAATGCCC GCATTCGGCTGCAGTTCA

RFC1 CATCGCCACCTTTCAGATT CCCGAAGACCAGGGCACAGA TGGCAAAGAACGTGTTGAC

RRM1 ACTAAGCACCCTGACTATGCTATCC CAGCCAGGATCGCTGTCTCTAACTTGCA CTTCCATCACATCACTGAACACTTT

RRM2 ACCGCGAGGAGGATCT TTTCGGCTCCGTGGGCTCCT TCAGCAGCGGCTCATC

TYMS GCCTCGGTGTGCCTTTCA TCGCCAGCTACGCCCTGCTCA CCCGTGATGTGCGCAAT

UMPS TAGTGTTTTGGAAACTGTTGAGGTT TGGCATCAGTGACCTTCAAGCCCTCCT CTTGCCTCCCTGCTCTCTGT

ACTB GAGCGCGGCTACAGCTT ACCACCACGGCCGAGCGG TCCTTAATGTCACGCACGATTT

for Japanese CRC series

ECGF1 GGATTCAATGTCATCCAGAG no probe CCTCCACGAGTTTCTTACTG

GGH AACCTCTGACTGCCAATTTCCATAA no probe TCTCTGGATGCCACTGGACAC

RRM2 CCCGCTGTTTCTATGGCTTC no probe CCCAGTCTGCCTTCTTCTTG

ACTB ATTGCCGACAGGATGCAGA no probe GAGTACTTGCGCTCAGGAGGA

**Supplementary Table 2.** Associations between mRNA expression and clinicopathological features in the Australian CRC series

Tumor site TILS *BRAF* mutation

Genes Proximal Distal p-value present absent p-value present absent p-value

CDA 2.08 (1.04 – 3.75) 2.42 (0.89 – 4.80) 0.685 2.06 (1.10 – 3.07) 2.21 (0.97 – 5.27) 0.479 3.81 (1.41 – 4.75) 2.12 (0.97 – 4.20) 0.426

DCK 2.55 (1.73 – 3.07) 2.59 (1.80 – 3.01) 0.945 2.90 (2.55 – 4.16) 2.53 (1.69 – 2.96) 0.013 2.70 (2.64 – 3.63) 2.54 (1.71 – 3.05) 0.088

DCTD 3.97 (2.96 – 5.02) 4.28 (3.27 – 5.63) 0.160 4.39 (3.92 – 4.95) 4.07 (3.05 – 5.29) 0.648 4.01 (3.36 – 4.77) 4.08 (3.01 – 5.33) 0.731

DHFR 3.74 (2.82 – 4.74) 4.39 (2.95 – 5.70) 0.335 4.52 (3.24 – 5.95) 3.78 (2.89 – 5.16) 0.181 4.75 (3.68 – 6.87) 3.90 (2.92 – 5.37) 0.151

DPYD 0.36 (0.29 – 0.59) 0.30 (0.23 – 0.46) 0.086 0.62 (0.30 – 0.94) 0.32 (0.25 – 0.47) 0.036 0.59 (0.30 – 1.07) 0.32 (0.25 – 0.49) 0.136

DUT 123.2 (67.6 – 169.0) 117.2 (68.0 – 166.4) 0.744 166.3 (87.1 – 198.6) 112.7 (67.8 – 162.2) 0.151 126.3 (100.7 – 198.6) 114.6 (64.4 – 166.1) 0.171

ECGF1 3.44 (2.58 – 4.94) 2.50 (1.85 – 4.03) 0.014 5.29 (3.46 – 7.38) 2.85 (1.96 – 4.17) 0.002 6.73 (3.99 – 8.17) 2.96 (2.04 – 4.17) 0.001

FOLR1 0.00 (0.00 –0.22) 0.16 (0.00 – 0.75) 0.008 0.15 (0.00 – 0.55) 0.08 (0.00 – 0.36) 0.612 0.00 (0.00 – 0.19) 0.08 (0.00 – 0.48) 0.422

FPGS 0.67 (0.53 – 0.84) 0.73 (0.50 – 0.95) 0.715 0.76 (0.50 – 0.88) 0.70 (0.52 – 0.87) 0.691 0.73 (0.60 – 0.86) 0.70 (0.52 – 0.89) 0.855

GGH 2.44 (1.38 – 4.45) 3.91 (2.32 – 5.22) 0.021 1.38 (1.16 – 2.52) 3.35 (2.08 – 5.55) 0.001 1.38 (0.94 – 2.27) 3.33 (2.08 – 5.84) 0.002

MTHFD1 3.94 (2.96 – 5.21) 4.06 (3.06 – 5.23) 0.623 4.15 (3.66 – 5.18) 3.98 (2.93 – 5.04) 0.485 4.68 (3.96 – 5.41) 3.87 (2.61 – 5.17) 0.136

MTHFR 1.12 (0.73 – 1.39) 0.85 (0.65 – 1.25) 0.189 1.28 (0.94 – 1.36) 0.94 (0.65 – 1.35) 0.081 1.32 (1.07 – 1.41) 0.94 (0.67 – 1.32) 0.027

RFC1 2.68 (1.93 – 3.43) 3.16 (2.14 – 4.38) 0.045 2.70 (2.14 – 3.65) 3.00 (92.00 – 4.05) 0.736 2.70 (1.82 – 4.09) 3.02 (2.12 – 4.05) 0.626

RRM1 0.94 (0.57 – 1.13) 0.99 (0.66 – 1.30) 0.295 0.99 (0.81 – 1.26) 0.95 (0.65 – 1.27) 0.407 1.07 (0.85 – 1.29) 0.96 (0.62 – 1.28) 0.204

RRM2 4.36 (2.23 – 6.53) 4.28 (2.39 – 6.80) 0.788 5.33 (3.69 – 7.27) 3.91 (2.28 – 6.39) 0.174 7.01 (4.70 – 7.650 3.91 (2.23 – 6.23) 0.030

TYMS 3.35 (2.27 – 4.28) 2.69 (1.95 – 4.29) 0.350 4.74 (3.71 – 5.87) 2.64 (1.92 – 4.00) 0.0002 4.48 (2.83 – 6.07) 2.99 (1.94 – 4.24) 0.029

UMPS 1.14 (0.90 – 1.52) 1.36 (1.05 – 1.98) 0.021 1.13 (0.85 – 1.59) 1.29 (1.02 – 1.72) 0.340 1.13 (1.09 – 1.44) 1.30 (0.99 – 1.750) 0.597

mRNA expression levels are presented with median (25th percentile – 75th percentile).

Mann-Whitney's U test was used for statistical analyses.
